# Sexual dysfunction in Brazilian patients with multiple sclerosis

**DOI:** 10.1055/s-0043-1767824

**Published:** 2023-05-09

**Authors:** Elisa Matias Vieira de Melo, Vinicius Andreoli Schoeps, Flavia Fairbanks Lima de Oliveira, Maria Fernanda Mendes, Guilherme Sciascia do Olival

**Affiliations:** 1 Santa Casa de São Paulo, Faculdade de Ciências Médicas, São Paulo SP, Brazil.; 2 Universidade de São Paulo, Faculdade de Medicina, Departamento de Ginecologia, São Paulo SP, Brazil.

**Keywords:** Multiple Sclerosis, Sexuality, Sexual Dysfunction, Physiological, Esclerose Múltipla, Sexualidade, Disfunções Sexuais Fisiológicas

## Abstract

**Background** People with multiple sclerosis (PwMS) show an increased risk of sexual dysfunction (SD), both in women and men.

**Objective** The aim of the present study was to apply the Multiple Sclerosis Intimacy and Sexuality Questionnaire-19 (MSISQ-19) and evaluate our results by comparing them with those in in the literature, as well as to assess the ease of applying the scale and the engagement of the patients in discussing the topic of sexuality.

**Methods** We developed and applied a web-based Google form questionnaire that the respondents completed online, which included the MSISQ-19, for the assessment of sexual function. Baseline characteristics were reported as proportions and mean ± standard deviation (SD) or median ± interquartile range (IQR) as appropriate according to data distribution. Categorical variables were stratified by sex and compared with chi-squared tests. Statistical analyses were performed using STATA v. 16 (StataCorp., College Station, TX, USA).

**Results** Of the 621 respondents, 541 were included in the analysis. Among the patients with MS, a total of 347 (64.14%) exhibited SD. When stratified by gender, the frequencies of SD were not significantly different.

**Conclusion** There is a high incidence of sexual dysfunction among PwMS and we need to identify the reasons for this and implement strategies to treat and counsel our patients. The MSISQ-19 can be used to help clinicians to assess sexual functioning in a quick and easy way and give patients the possibility to address this topic and receive appropriate help and support.

## Resumo

**Antecedentes** Pacientes com esclerose múltipla apresentam altas taxas de disfunção sexual em diversos estudos.

**Objetivo** Avaliar a aplicação e resultados da ferramenta para avaliação de disfunção sexual em pacientes com Esclerose Multipla, Multiple Sclerosis Intimacy and Sexuality Questionnaire-19 (MSISQ-19), bem como comparar os resultados encontrados com a literatura já existente e ter a percepção do engajamento dos pacientes sobre o tema.

**Métodos** Realizado através de questionário on-line na plataforma Google Forms, composto por questões sobre perfil demográfico e relacionadas à doença e o questionário MSISQ-19. Os dados demográficos e sobre a doença foram descritos como proporções e média ± desvio padrão (DP) ou mediana ± intervalo interquartil (IIQ). As variáveis categóricas foram estratificadas por sexo e comparadas com testes de qui-quadrado. As análises estatísticas foram realizadas no programa STATA v. 16 (StataCorp., College Station, TX, EUA).

**Resultados** Um total de 621 pacientes responderam ao questionário, sendo inclusos na análise 541 respostas, após a aplicação dos critérios de elegibilidade. Um total de 347 (64,14%) pacientes apresentaram disfunção sexual, sem diferença entre os gêneros.

**Conclusão** Disfunção sexual apresenta alta prevalência no grupo estudado, sendo necessário identificar as causas e implementar estratégias de tratamento. O MSISQ-19 é uma ferramenta fácil e rápida de ser aplicada, podendo ser utilizada para facilitar a comunicação com os pacientes sobre o tema, possibilitando assim o tratamento das disfunções sexuais nessa população.

## INTRODUCTION

Multiple sclerosis (MS) is a chronic inflammatory disease of the central nervous system which is characterized by inflammation, demyelination, axon loss and gliosis 1 and afflicts > 2.8 million people worldwide. 2 It is most commonly diagnosed in young people of reproductive age and has significant lifelong repercussions. 3 Among the many MS symptoms, sexual dysfunction (SD) is one that is commonly overlooked during routine clinical care and is comprised of three main aspects 4 5 6 7 8 :

- Primary (SD1): refers to the direct result of neurological injuries involving the genital system itself;
- Secondary (SD2): refers to physical disorders related to MS and the side effects of drugs that indirectly influence the sexual response;
- Tertiary (SD3): refers to the consequences of the cultural, social, emotional, and psychological effects of MS.

A recent systematic review showed that the rate of events of SD on average in patients with MS and healthy controls was 70.9 and 49.9%, respectively; the absolute effect was 434 more per 1,000. 8 A study conducted by Silva et al. 6 showed that among patients with MS, 54.3% of males and 71.7% of females presented some kind of SD; in the control group the results were 12.5 and 19.5%, respectively. A review demonstrated that people with multiple sclerosis (PwMS) have similar rates of SD are in both sexes (33–75% in women and 47–75% in men). 9

The aim of the present study was to apply the Multiple Sclerosis Intimacy and Sexuality Questionnaire-19 (MSISQ-19) and compare our results with those in the literature, applicability, and engagement of PwMS to talk about SD in Brazil.

## METHODS

The present cross-sectional study was carried out at the Santa Casa de São Paulo, Faculdade de Ciências Médicas, São Paulo, state of São Paulo, Brazil, between January and August 2020.

Considering the sensitivity of the subject, we developed and applied a web-based Google Forms questionnaire in order to assess the characteristics and sexual function of the patients. People with multiple sclerosis were invited to participate via e-mail after an in-person consultation at the Santa Casa de São Paulo's Neuroimmunology clinic or through contacting individuals on a mailing list obtained from the Brazilian MS Patient Association. Respondents self-reported their MS diagnosis, concurrent medical conditions, drug use, demographics, and completed the MSISQ-19 for the evaluation of their sexual activity and satisfaction over the previous 6 months.

The MSISQ-19 is a self-report tool that measures sexual dysfunction and has been validated in the MS population. It comprises 19 questions with each item scored as 1 (never), 2 (seldom), 3 (sometimes), 4 (often) and 5 (always). Responses of 4 (often) or 5 (always) indicate SD. 6 10 11 The MSISQ-19 also provides scores for three different subscales: primary sexual dysfunction – SD1 (items 12, 16, 17, 18, and 19), secondary sexual dysfunction – SD2 (items 1, 2, 3, 4, 5, 6, 8, 10, and 11) and tertiary sexual dysfunction – SD3 (7, 9, 13, 14, and 15). The original English tool was developed in 2000 11 and subsequently validated in Brazilian/Portuguese 6 see the ( Supplementary Material ).

The inclusion criteria were respondents who self-reported as having MS, agreed to participate and were able to answer the questionnaire online, irrespective of age and sex, and there was no requirement for a minimum number of responses to the MSISQ-19 online. The exclusion criteria were individuals who provided duplicate answers or failed to respond to any of the 19 statements.

Baseline characteristics were reported as proportions and mean ± standard deviation (SD) or median ± IQR as appropriate according to data distribution. Categorical variables were stratified by sex and compared with chi-squared tests. Two-sided p-values < 0.05 were considered significant without correction for multiple comparisons considering the exploratory study design. Scores were calculated for the MSISQ-19 with the data provided irrespectively of the degree of missingness, no imputation procedure was performed. Statistical analyses were performed using STATA v. 16 (StataCorp., College Station, TX, USA).

The study protocol was approved by Santa Casa de Sao Paulo's Institutional Review Board (number 33471420.4.0000.5479). Informed consent was given online and was required before taking the survey.

## RESULTS

Of the 621 respondents, 541 were included in the analysis after applying the eligibility criteria described above. The sociodemographic and clinical data are shown in Table 1 .

**Table 1:** Descriptive characteristics

Characteristics		*n* (%)
Age in years, mean (SD)		37.0 (9.9)
Sex	Female, *n* (%)	445 (82.3)
	Male, *n* (%)	92 (17.0)
Age at disease onset, mean (SD)		28.2 (9.4)
Time since diagnosis	0 to 3 years	218 (40.3)
	4 to 9 years	185 (34.2)
	≥ 10 years	131 (24.2)
	No answer	7 (1.3)
Self-Informed ethnicity	White	364 (67.3)
	Black	26 (4.8)
	Multiracial	144 (26.6)
	Yellow	4 (0.7)
	Indigenous	0 (0)
	No answer	3 (0.6)
Schooling	0 to 8 years	9 (1.7)
	9 to 11 years	146 (27.0)
	> 12 years	385 (71.2)
	No answer	1 (0.2)
Occupation	Employed	229 (42.3)
	Self-employed	75 (13.9)
	Unemployed	72 (13.3)
	Retired	90 (16.6)
	Homemaker	23 (4.6)
	Other (e.g., student, medical license)	47 (8.7)
	No answer	5 (0.9)

Abbreviation: SD, standard deviation.

Among the patients with MS, a total of 347 (62.2% of the male individuals and 63.8% of the females) exhibited some degree of SD. Considering the subscales, the most predominant was SD2 (51.9%), followed by SD1 (46%) and SD3 (44%). When stratified by gender, the frequencies of global SD and the SD1, SD2, and SD3 subscales were not significantly different.

The median total MSISQ-19 score was 40 (28–56); in the subgroups, the median score was 52 (40-62) in PwMS with SD and 25 (22–32) in PwMS without SD.

According to responses regarding SD1, which relates to primary causes of sexual dysfunction (lack of sexual desire, orgasm problems and lubrication/erectile problems), we evaluated the results according to gender. The most common issues reported by men were orgasm problems (54.2%) followed by erectile dysfunction (33.3%). In women, it was orgasm problems (66.3%) and lubrification difficulties (32.8%). Comparing complaints between genders, our results show a statistically significant difference in relation to lack of desire, with this complaint being more frequent among women ( Table 2 ).

**Table 2:** Association between sex and sexual dysfunction type 1 questions, *n* (%)

Sexual dysfunction type 1 questions	Total sample	Female	Male	*p* -value
Less feeling or numbness in my genitals	120 (23.2)	95 (22.4)	24 (26.7)	0.38
Lack of sexual interest or desire	149 (23.4)	129 (29.9)	17 (19.1)	0.04*
Less intense or pleasurable orgasms or climaxes	157 (30)	129 (30)	25 (28.1)	0.72
Takes too long to orgasm or climax	182 (35)	156 (36.3)	23 (26.1)	0.07
Inadequate vaginal wetness or lubrication (women)/ difficulty getting or keeping a satisfactory erection (men)	171 (32.8)	141 (32.8)	29 (33.3)	0.92

Notes: *statistically significant difference.

Analyzing subtype 2 SD, which deals with symptoms related to MS and its interference in sexual satisfaction, we observed that cognitive deficits represented the most common complaint (32.8%), followed by impaired mobility (23.1%) and pain (20.2%). There were no statistically significant differences related to any symptoms between females and males ( Table 3 ).

**Table 3:** Association between sex and sexual dysfunction type 2 questions, *n* (%)

Sexual dysfunction type 2 questions	Total sample	Female	Male	*p* -value
Muscle tightness or spasms in my arms, legs, or body	87 (16.7)	73 (17)	13 (14.4)	0.55
Bladder or urinary symptoms	81 (15.5)	66 (15.4)	14 (15.4)	0.99
Bowel symptoms	61 (11.8)	51 (12)	9 (10.1)	0.62
Feelings of dependency because of MS	87 (16.9)	71 (16.8)	14 (15.7)	0.80
Tremors or shaking in my hands or body	77 (14.8)	57 (13.44)	19 (20.65)	0.08
Pain, burning, or discomfort in my body	106 (20.2)	90 (21)	15 (16.5)	0.33
Problems moving my body the way I want during sexual activity	121 (23.1)	102 (23.7)	18 (20.2)	0.48
Problems with concentration, memory, or thinking	173 (32.8)	148 (34.2)	23 (25.3)	0.10
Exacerbation or significant worsening of my MS	63 (12.4)	50 (12)	12 (13.5)	0.69

Abbreviation: MS, multiple sclerosis.

Analyzing subtype 3 SD, the 2 most important concerns related to feeling less attractive (30.1%) and worrying about sexually satisfying his/her partner (28.1%). There were no significant differences between females and males ( Table 4 ).

**Table 4:** Association between sex and sexual dysfunction type 3 questions n (%)

Sexual dysfunction type 3 questions	Total sample	Female	Male	*p* -value
Feeling that my body is less attractive	156 (30.1)	126 (29.7)	29 (32.2)	0.63
Feeling less masculine or feminine due to MS	96 (18.5)	80 (18.8)	16 (18)	0.85
Fear of being rejected sexually because of MS	115 (22.3)	91 (21.6)	22 (24.2)	0.59
Worries about sexually satisfying my partner	152 (29.4)	119 (28.1)	30 (33.7)	0.29
Feeling less confident about my sexuality due to MS	137 (26.6)	114 (27)	22 (24.7)	0.66

Abbreviation: MS, multiple sclerosis.

Considering the cumulative frequencies of SD types, we observed that in 76.1% of patients with sexual dysfunction, there are positive results in ≥ 2 SD subscales (23.9%, 1 sexual dysfunction type; 30.8%, 2 sexual dysfunction types; and 45.3%, 3 sexual dysfunction types).

There was no association between disease length and a higher prevalence of SD (odds ratio [OR]: 0.99 (0.96–1.01); *p* = 0.22).

Considering age and SD, when a stratified analysis by median age was performed, an association was found between MS patients > 40 years old and increased rates of SD (OR: 1.44 (0.99-2.09); *p* = 0.059).

## DISCUSSION

Sexual health is recognized as being an important factor in long-term relationships and quality of life. The pleasurable aspect of sex has a greater importance than its purely reproductive purpose for both women and men, although some factors limit discussions about sexuality. Sexual dysfunction can be the result of sociocultural, physiological, biological, psychological, and couple relationship determinants. A large proportion of people with SD do not seek medical help because of frustration, shame, or the failure of previous treatment attempts. Moreover, there is limited clinical research showing positive results for interventions in sexual dysfunctions of organic origin, a lack of effective treatments for female SD and insufficient evidence of the efficacy of drugs or of psychological interventions. 12

Sexual dysfunction is defined by the World Health Organization (WHO) as not being able to participate as desired in a sexual relationship. It includes biological, psychosocial, and interpersonal problems such as loss of sexual desire, sexual aversion, lack of enjoyment, erectile dysfunction, lack of lubrication, orgasmic dysfunction, premature ejaculation, vaginismus, dyspareunia or pelvic pain, substance- or medication-induced dysfunction, post-orgasmic illness, persistent genital arousal, and hypersexuality. 9 10 11 12 13 14 15 16 The estimated prevalence of SD is of ∼ 40 to 45% in women and of ∼ 20 to 30% in men in the general population. 17

The MSISQ-19 is a questionnaire that can easily be applied during any patient consultation. It is a self-reported questionnaire and should be adapted and validated to take into consideration the cultural differences of the country in which it is used. Many studies have incorporated the MSISQ-19 as a primary measure of SD in MS. 6 18

Regarding MS, there are several studies demonstrating that SD is prevalent in PwMS, 6 8 9 10 19 as was found in our study. However, our results, unlike those from other studies, showed complaints about orgasmic dysfunction in both genders, not just in women. 17 18 20 Our results were in agreement with other studies regarding women with MS reporting hypoactive sexual desire more frequently. 21 22 23 24 25

Considering the three subtypes of SD described in patients with MS (SD1, SD2, and SD3), we observed that participants may present multiple dysfunctions at the same time. In addition, > 50% of the sample reported dysfunction in subscale 2 (SD2), indicating that the symptoms/sequelae of MS interfere in sexual intercourse. 26 27 Among these symptoms/sequelae, those which appear to interfere more significantly are cognitive deficits, impaired mobility, and pain. Problems with memory related to cognitive impairment are a particular issue, and issues with depression and anxiety, reflected by the high scores in these scales, may also play an important role. 28 29 30 According to Valleroy et al., symptoms such as muscle weakness, spasticity, and incontinence are those most frequently responsible for sexual impairment, which are divergent from our findings. 31

According to some studies, the duration of the disease was also significantly associated with SD 26 32 ; however, this finding was not confirmed by our study, which is in agreement with a number of other studies. 33 34 The relationship between SD and disease duration is, therefore, unclear. Aging has also been identified as a risk factor for the development of SD. 6 8 25 35 In our study, being > 40 years old was positively associated with SD, while no association was found in those aged < 40 years old. These results suggest that sexual function declines with age in PwMS, although we need to understand the correlation between this data and disease, comorbidities, hormonal changes, and other factors that may affect sexuality.

Due to the high prevalence of SD presented in our study, which has been corroborated by several other studies previously mentioned, it is essential to include the theme of sexuality during routine consultations from the initial diagnosis. According to data from McCabe et al., 36 95% of PwMS have never been asked about their sexual functioning. Lew-Starowicz et l. 37 conducted a study which enrolled 137 MS patients, and only 2.2% of them reported having been asked about their sexual function by their physicians. This may be partly explained by the limited time available for clinical assessment and consultation, but it may also be the result of the difficulties associated with approaching this topic, both from the perspective of the clinician and of the patient. The use of self-reported questionnaires that can be completed by the patient in privacy, for example just before the clinical consultation begins or online, can offer a profile of the sexual performance of the patient.

In our study, we used the internet as a tool for applying the MSISQ-19 questionnaire, which had been validated for use in Portuguese. In this way, pre-existing scales can be used virtually, via email or a link, to enable the remote application of these scales. The benefits of this method of measurement can be seen through the three major strengths of this technique. First, confidential surveys are well suited to capture experiences, perceptions, and attitudes of individuals. Second, the same pre-existing scales can be used across different studies, enabling the comparison and replication of the results and the application of these types of survey allows data to be collected from large samples at a relatively low cost, and they can produce generalizable results, which provide an understanding of the views or experiences of a population group. Third, the validity and reliability of survey instruments can be assessed through rigorous, transparent, and well-accepted validation methods, providing the researcher with confidence that the measures are relevant and accurate. 38 Although some studies reported some problems with online tools, such as noncompletion of the questionnaire 39 and despite the fact that the questionnaire requires some attention and commitment to satisfactorily answer the 19 questions, we had good engagement, demonstrating the interest and concern of the patients in relation to these issues, and reinforcing the need for routine discussion about sexuality.

Our study has some limitations that should be considered. As our sample relies on self-report and consists of patients who completed the questionnaire online, it may not be possible to compare the results with clinical data regarding factors such as neurological examinations, MS types, the expanded disability status scale (EDSS), and the use of medications (antidepressive drugs, anticonvulsant drugs, muscle relaxant, etc.). In addition, the fact that the study was conducted through the Internet may have created a bias regarding the higher education among the interviewees (reflected by the high percentage of participants with higher or postgraduate education), and it is not possible to measure the real impact of schooling on the rate of sexual dysfunction. Moreover, as the study was undertaken during the COVID-19 pandemic, we cannot identify whether this influenced levels of SD, since no evaluation of SD was performed in a control group. Another possible limitation is the use of a scale developed for patients with MS, without scales of sexual dysfunction applied in the general population being used, which would allow comparison of the results with people without the disease. To address this, our group is currently conducting a study with PwMS and a group of healthy controls.

In conclusion, good sexual health is fundamental to the quality of life of all individuals, including those with MS, and must be included in discussions about the disease, particularly in consultations, but also in all other forums and education strategies. There is a high incidence of sexual dysfunction among PwMS and we need to identify the reasons for this and implement strategies to treat and counsel our patients. The MSISQ-19 can be used to help clinicians to assess sexual functioning in a quick and easy way and give patients the opportunity to address this topic and receive appropriate help and support. Further studies are required to better understand the variables that affect and impact sexuality in PwMS.
