# Supplementary material for: Sexual dysfunction in Brazilian patients with multiple sclerosis
Source: Arq Neuropsiquiatr. 2023 May 9;81(4):350–6. doi: 10.1055/s-0043-1767824 (PMC10169235; doi:10.1055/s-0043-1767824)
Supplement: Supplementary file 1 — Supplementary Material [file 10-1055-s-0043-1767824-s220157.pdf]

Supplementary Material

S1: Multiple Sclerosis Intimacy and Sexuality Questionnaire-19 [MSISQ-19].

Instructions

In order to better understand the impact of multiple sclerosis (MS) on intimacy and sexuality, this 19-item questionnaire asks you to rate how various MS symptoms have interfered with your sexual activity or satisfaction over the last 6 months. Questions may be answered by placing a check or any other mark in the square located next to the question and below the appropriate number. There are no right or wrong answers. If you are unsure how to answer a question, please choose the best answer you can.

Over the last 6 months, the following symptoms have interfered with my sexual activity or satisfaction:	Never	Rarely	Occasionally	Almost always	Always
	1	2	3	4	5
1. Muscle tightness or spasm in my Arms, legs, or body					
2. Bladder ou urinary symptoms					
3. Bowel symptoms					
4. Feelings of dependency because of MS					
5. Tremors or shaking in my hands or body					
6. Pain, burning, or discomfort in my body					
7. Feeling that my body is less attractive					
8. Problems moving my body the way I want during sexual activity					
9. Feeling less masculine or feminine due to MS					
10. Problems with concentration, memory, or thinking					
11. Exacerbation or significant worsening of my MS					
12. Less feeling or numbness in my genitals					
13. Fear of being rejected sexually because of MS					
14. Worries about sexually satisfying my partner					
15. Felling less confident about my sexuality due to MS					
16. Lack of sexual interest or desire					
17. Less intense or pleasurable orgasms or climaxes					
18. Takes too long to orgasm or climax					
19. Inadequate vaginal wetness or lubrification (women)/ difficulty getting or keeping a satisfactory erection (men)					

Scoring:
Primary sexual dysfunction subscale items = 12, 16, 17, 18, 19
Secondary sexual dysfunction subscale items = 1, 2, 3, 4, 5, 6, 8, 10, 11
Tertiary sexual dysfunction subscale items = 7, 9, 13, 14, 15

Any item scoring "4" or "5" should be discussed with your MS healthcare professional

Source: Silva RAP, Olival GS, Stievano LP, Toller VB, Jordy SS, Eloi M, et al . Validation and cross-cultural adaptation of sexual dysfunction modified scale in multiple sclerosis for Brazilian population. Arq Neuro-Psiquiatr. 2015;73(8):681–7.

S2: Multiple Sclerosis Intimacy and Sexuality Questionnaire-19 [MSISQ-19] – Brazilian version. [Escala sobre intimidade e sexualidade de 19 itens na Esclerose Múltipla - Multiple Sclerosis Intimacy and Sexuality Questionnaire-19 (MSISQ-19) - validado em português (2015)].

Instruções

Para melhor entender o impacto da esclerose múltipla (EM) na intimidade e sexualidade, este questionário com 19 itens pede para você classificar como os diversos sintomas da EM têm interferido na sua atividade sexual ou na sua satisfação sexual nos

 © 2023. Academia Brasileira de Neurologia. All rights reserved.

últimos 6 meses. As questões podem ser respondidas através de uma marcação no quadrado localizado ao lado da questão e abaixo do número que correspondente à sua resposta. Não existe resposta certa ou errada. Caso você não tenha certeza de como responder à pergunta, por favor, escolha a melhor resposta que você puder.

Nos últimos 6 meses, os sintomas abaixo têm interferido na minha atividade sexual e satisfação sexual	Nunca	Quase nunca	Ocasionalmente	Quase sempre	Sempre
	1	2	3	4	5
1. Rigidez muscular nos meus braços, pernas ou corpo					
2. Sintomas de incontinência urinária					
3. Sintomas no intestino					
4. Sentimentos de dependência por causa da EM					
5. Tremores ou agitações em minhas mãos e corpo					
6. Dor, queimação ou desconforto em meu corpo					
7. Sentimento de que meu corpo é menos atrativo					
8. Problemas movendo meu corpo do jeito que eu quero durante a atividade sexual					
9. Sentindo-me menos masculino ou feminino devido à EM					
10. Problemas de concentração, memória ou pensamentos					
11. Exacerbação ou significante agravamento da minha EM					
12. Menor sensibilidade ou entorpecimento das minhas genitais					
13. Medo de ser rejeitado sexualmente por causa da EM					
14. Preocupações a respeito da satisfação sexual do meu parceiro					
15. Sentindo-me menos confiante sobre minha sexualidade devido à EM					
16. Falta de desejo ou interesse sexual					
17. Orgasmo ou clímax menos intensos e/ou menos prazerosos					
18. Demora ao orgasmo ou clímax					
19. Lubrificação e umidade vaginal inadequada (mulher)/ dificuldade de obter ou manter ereção satisfatória (homem)					

Escore:
Disfunção sexual primária = questões 12, 16, 17, 18, 19
Disfunção sexual secundária = questões 1, 2, 3, 4, 5, 6, 8, 10, 11
Disfunção sexual terciária = questões 7, 9, 13, 14, 15

Qualquer resposta "4" ou "5" deve ser discutida com o profissional de saúde.
Source: Silva RAP, Olival GS, Stievano LP, Toller VB, Jordy SS, Eloi M, et al. Validation and cross-cultural adaptation of sexual dysfunction modified scale in multiple sclerosis for Brazilian population. Arq Neuro-Psiquiatr. 2015;73(8): 681–7.

 © 2023. Academia Brasileira de Neurologia. All rights reserved.
